# Triglyceride-to-high density lipoprotein cholesterol ratio predicts clinical outcomes in patients with gastric cancer

**DOI:** 10.7150/jca.35939

**Published:** 2019-11-01

**Authors:** Huiling Sun, Xiaoqin Huang, Zemu Wang, Guoxing Zhang, Yanping Mei, Yishan Wang, Zhenlin Nie, Shukui Wang

**Affiliations:** 1 General Clinical Research Center, Nanjing First Hospital, Nanjing Medical University, Nanjing 210006, China; 2 Department of Clinical Laboratory, Nanjing First Hospital, Nanjing Medical University, Nanjing 210006, China; 3 Department of Cardiology, The First Affiliated Hospital of Nanjing Medical University, Nanjing 210029, China

**Keywords:** triglyceride, high density lipoprotein cholesterol, gastric cancer, clinical outcome, nomogram

## Abstract

Correlation of triglyceride (TG)-to-high density lipoprotein cholesterol (HDL-C) ratio (TG/HDL-C) and the survival of gastric cancer (GC) remain unclear. The purpose of this study was to explore the precise effect of preoperative TG/HDL-C on clinical outcomes in GC patients. Patients with GC were enrolled from 2006 to 2014. A total of 957 individuals from a single center were divided into prospective training and retrospective test cohorts. The optimal cutoff value of TG/HDL-C was determined using X-tile software to separate the training cohort into low and high survival groups according to TG/HDL-C levels. Survival analyses were performed using Kaplan-Meier curves and a Cox proportional hazards regression model. Preoperative TG/HDL-C and clinical outcomes were obtained to determine the prognostic significance of serum lipids in the training and test cohorts. We observed that high TG and TG/HDL-C were significantly correlated with poor outcome in GC patients, and high TG/HDL-C harbored the highest area under curve to independently predict 5-year overall survival in two cohorts. Furthermore, c-index of the prognostic nomogram including TG/HDL-C was significantly higher than that without it. In summary, TG/HDL-C was an efficient and independent prognostic factor to predict 5-year case fatality of GC patients and to improve the efficacy of its prognostic nomogram.

## Introduction

Gastric cancer (GC) is one of the most common gastrointestinal tumors worldwide. There are more than 70% of GC patients in developing countries of Eastern Asia and China 1. GC is the second most common tumors and third leading cause of death in 2015 according to Chinese Cancer Statistics 2. Despite substantial development obtained in diagnosis and therapy for GC in recent years, the survival rate remains unsatisfactory because of tumor metastasis and relapses after surgery. The prognosis of GC patients may be improved with the detection of GC at its early stage 3. Therefore, it is essential to find biomarkers to detect early stage of GC or predict clinical outcomes in individual patients with GC.

Dyslipidemia has been reported to be associated with various diseases including GC 4. It is all known that dyslipidemia is significantly correlated with obesity, and obesity has been associated with increased risk of poor outcome in GC patients 5. Triglyceride has served as an independent indicator for fatty acid oxidation involving tumor cell proliferation and growth 6. However, the association between triglyceride (TG) and GC development remains unclear. Additionally, cholesterol has been reported to be involved in GC progression 7. Conversely, high density lipoprotein cholesterol (HDL-C) presents anti-inflammatory capacity 8, which has been inversely correlated with GC risk 4. A previous study showed that TG to HDL-C ratio (TG/HDL-C) was superior to TG as a predictor for the survival of triple negative breast cancer patients 9. Another study of the meta-analysis reported the protective factors of total cholesterol (TC) and HDL-C for overall survival in cancer patients 10. However, the association of preoperative TG/HDL-C with GC survival remained unclear.

In the present study, we aimed to investigate whether preoperative serum lipid levels affect GC mortality in GC patients undergoing surgical resection. Additionally, we attempted to construct a prognostic nomogram to improve the predictive capacity for GC patients according to clinicopathologic parameters and significant biomarkers.

## Methods

### Study population

We reviewed clinical characteristics of GC patients. All patients were divided into two cohorts. A total of 572 patients who underwent surgical resection or chemotherapy were prospectively enrolled in the training cohort between January 2010 and November 2014. The test cohort retrospectively collected 385 patients who underwent surgical resection or chemotherapy between January 2006 and December 2009. All individuals were from Nanjing First Hospital, Nanjing Medical University. The inclusion and exclusion criteria were as follows: 1) each informed consent was obtained from GC patients; 2) patients did not underwent anticancer treatment before surgery; 3) patients underwent a curative or palliative gastrectomy with standard lymphadenectomy; 4) the estimated life expectancy ≥ 3 months after surgery; 5) all patients were verified with biopsy-proven gastric adenocarcinoma. The clinicopathological findings were determined according to the 7^th^ edition of gastric cancer tumor-node-metastasis (TNM) staging system of American Joint Committee.

All telephone or outpatient follow-ups of individuals performed as follows: occurred every 3 months for the first 2 years and then every half a year for 3 years, started after treatment and continued until January 2019 or until death. A total of 49 (5.12%) patients lost to follow-up during the follow-up period. The protocol was approved by the Medical Ethics Committee of Nanjing First Hospital, Nanjing Medical University. Informed consent for use of their information was obtained from all enrolled patients.

### Sample processing and laboratory detection

To explore the correlation of serum lipid levels (triglyceride [TG]; total cholesterol [TC]; low-density lipoprotein cholesterol [LDL-C]; TG to HDL-C ratio [TG/HDL-C]) and clinical outcomes of GC patients, preoperative peripheral blood, plasma and serum samples were collected from all enrolled patients at 5:00 to 8:00 am prior to surgery, and were detected within two hours after the sample collection. Preoperative serum lipid levels of blood samples were routinely tested by a blood analyzer (Beckman Coulter AU5841 [Beckman Coulter, CA, USA]). Additionally, *Helicobator pylori* infection was assessed by 13C-UBT.

### Statistical analysis

Continuous parameters were presented as mean ± standard deviation or median and inter-quartile ranger (IQR) if necessary. Categorical parameters were presented as number and percentage. Optimal cutoff values for serum lipid levels replying on 5-year overall survival (OS) were determined using X-tile 3.6.1 software (Yale University, New Haven, CT, USA) 11. Mann-Whitney U test was used to compare the difference of continuous parameters, and the difference of categorical parameters was compared by Chi-square or Fisher's exact test. Survival analysis was assessed by Kaplan-Meier curve and log-rank test. Cox proportional regression analysis was used to explore prognostic significance of clinical characteristics in GC patients. Predictive capacity of significant parameters was compared by receiver operating characteristic (ROC) curve and area under ROC curve (AUC). A prognostic nomogram was constructed according to clinical characteristics, and its predictive efficacy was assessed by Harrell's concordance index (c-index) and calibration curve. *P*<0.05 was considered as statistical significance. All data analyses were conducted using SPSS 20.0 version (SPSS Inc., Chicago, IL, USA) and R 3.3.1 software (Institute for Statistics and Mathematics, Vienna, Austria).

## Results

### Baseline characteristics of GC patients

Clinical characteristics for training and test cohorts are detailed in Table 1. A total of 572 patients with GC were prospectively collected in the training cohort. There were 302 (52.8%) patients with *helicobator pylori* infection, 202 (35.3%) with distant metastasis and 280 (49.0%) undergoing chemotherapy in the training cohort. A total of 32 patients dropped out of the follow-up, and 294 cases died during the follow-up period. An additional 385 GC patients were retrospectively enrolled in the test cohort. Of the test cohort, 48 (12.5%) cases had early gastric cancer, and 129 (33.5%) underwent distant metastasis. A total of 17 patients dropped out of the follow-up, 205 patients had died during the follow-up period.

### The optimal cutoff value of TG/HDL-C

**Figure 1:**
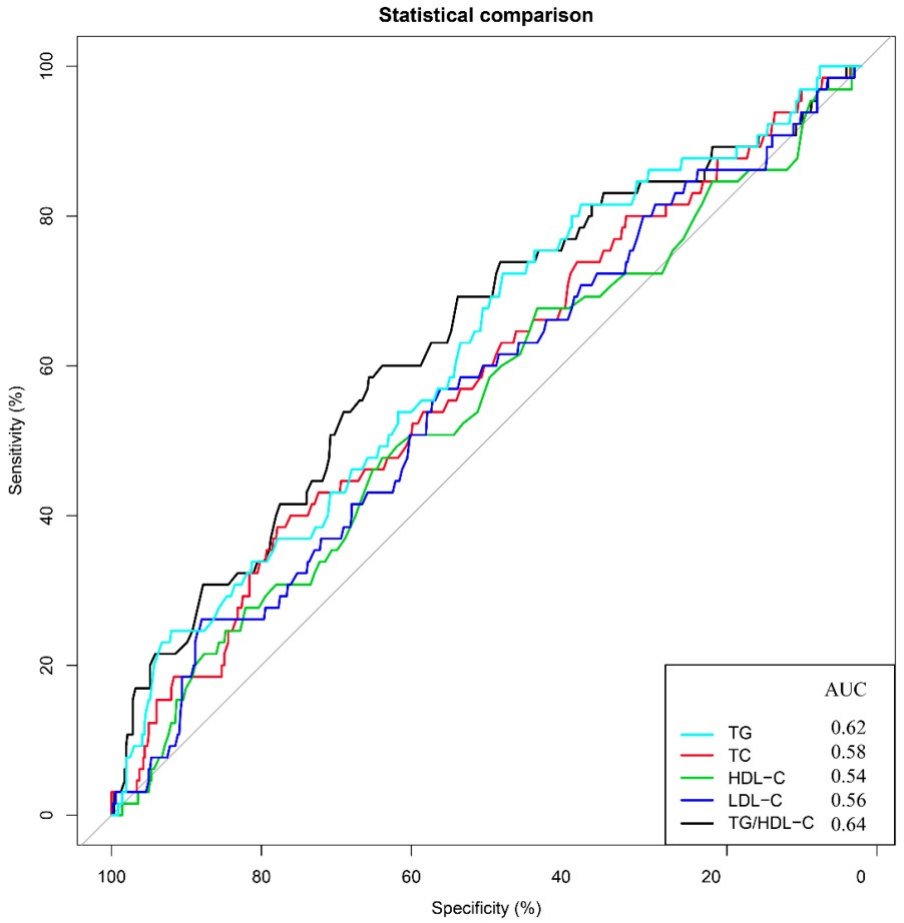
** Predictive values of TG, TC, HDL-C, LDL-C, and TG/HDL-C for 5-year overall survival.** Receiver operating characteristic curves and areas under the curves for overall survival: 0.62 for TG, 0.58 for TC, 0.54 for HDL-C, 0.56 for LDL-C and 0.64 for TG/HDL-C.

**Figure 2:**
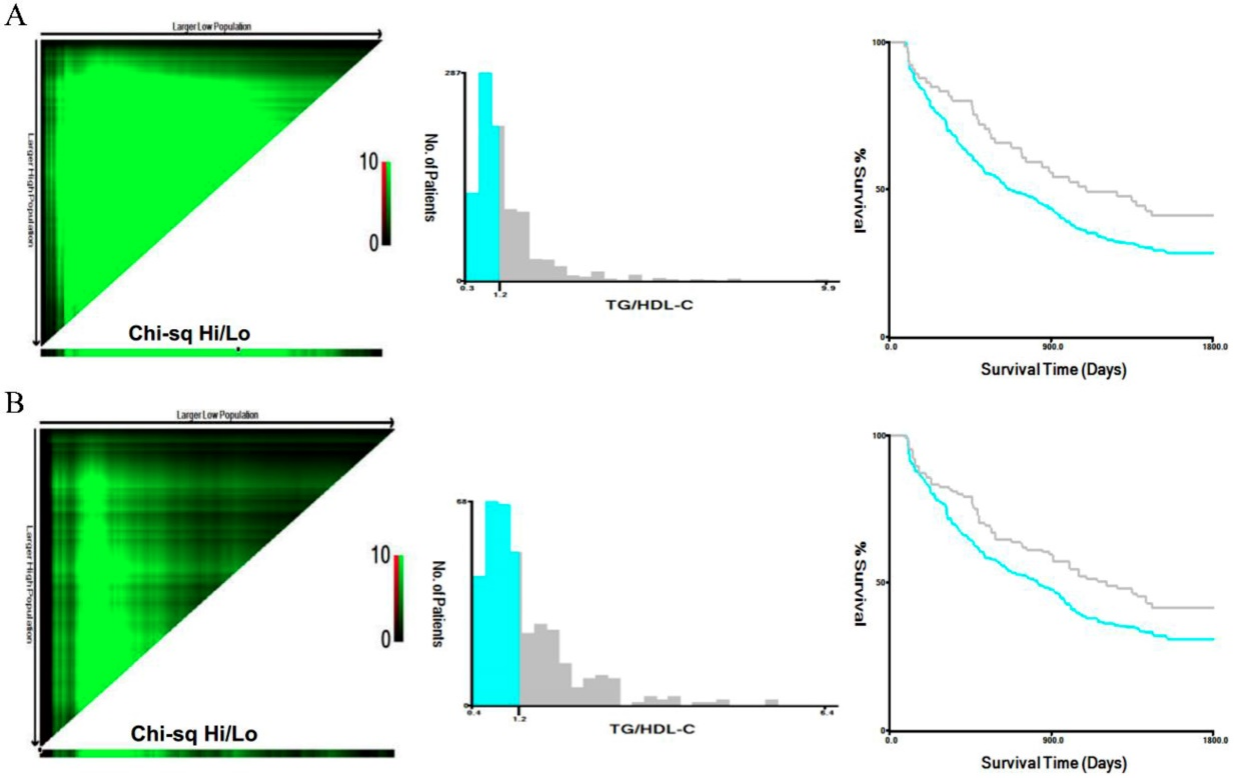
** Analysis of TG/HDL-C using X-tile.** The black circles highlight the optimal cutoff values which are presented in histograms in the training (A) and test (B) cohorts. Survival curves of TG/HDL-C in the training and test (B) cohorts. The high TG/HDL-C group had higher 5-year mortality rates than the low group in the training cohort of gastric cancer patients (*P*<0.001).

To assess the prognostic significance of preoperative lipid levels in GC patients, we performed ROC curve analyses to compare predictive capacity of TG, TC, HDL-C, LDL-C and TG/HDL-C in the training cohort (Figure 1). The results showed that the AUC value of TG/HDL-C was significantly higher than those of other serum lipid levels (all *P*<0.05). Subsequently, we used the X-tile to further determine the optimal threshold of TG/HDL-C (Figure 2). The results showed that the cutoff value of TG/HDL-C was 1.20 according to 5-year mortality in the training cohort patients. According to its cutoff value, the training cohorts were separated into high- and low-level groups. We then assessed the association of TG/HDL-C with clinical characteristics in the two cohorts (Table 2). In the training cohort, age (*P*=0.028) and depth of invasion (*P*<0.001) were significantly different between the high and low TG/HDL-C groups. Similar results were found when the test cohort was separated into two groups for clinical features according to the same cutoff value (Table 2).

### Correlation of TG/HDL-C with survival

We performed survival analyses to investigate the correlation of clinical features with clinical outcomes in the training and test cohorts (Table 3). Our results showed that patients with patients with advanced age, tumor differentiation (moderate/poor), and advanced TNM stage, palliative treatment, and increased TG and TG/HDL-C suffered lower 5-year survival rates (all *P*<0.05, Table 3 and Figure 2). Furthermore, age, TNM stage, TG, and TG/HDL-C were independent predictors for 5-year mortality in both two cohorts after adjustment (Table 3).

### Predictive capacity of TG/HDL-C for survival

**Figure 3:**
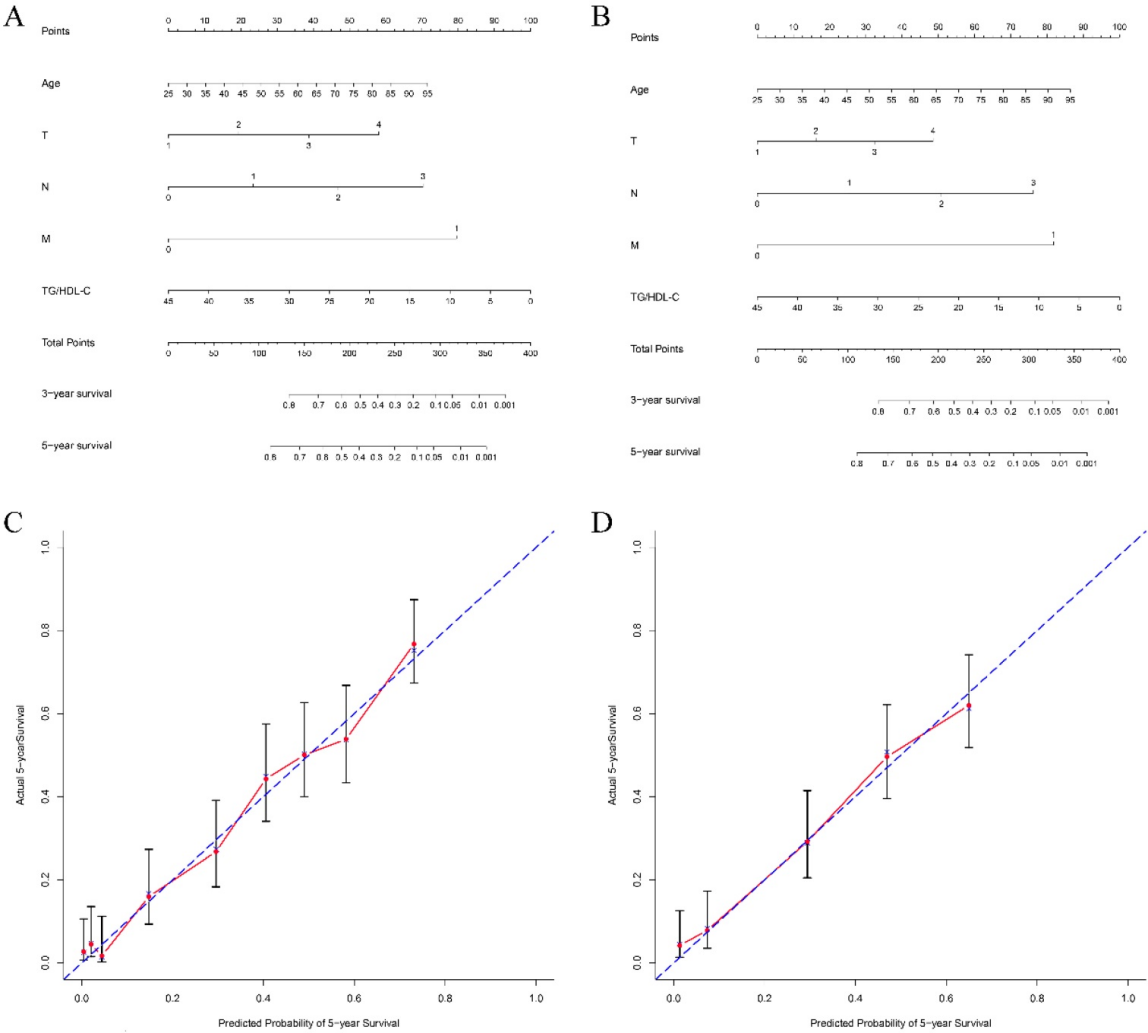
** Nomograms of patients with gastric cancer to predict 5-year overall survival.** Locate the TG/HDL-C on the respective axis; draw a straight line up to the Points axis to determine how many points toward 5-year overall survival the patient receives for the TG/HDL-C; repeat this process for other variables; add the points and locate this number on the Total points axis; and draw a straight line down to find the patient's estimated risk of 5-year overall survival. The c-indexes for the training and test cohorts of patients are 0.732 (A) and 0.725 (B), respectively. Calibration curves for overall survival, which are representative of predictive accuracy, for the training (C) and the test cohorts (D). The 45-degree reference line represents a perfect match between predicted and observed values.

To explore the predictive capacity of TG/HDL-C in GC patients, we constructed nomograms in terms of significant characteristics and TG/HDL-C. The prognostic nomograms with or without TG/HDL-C are shown in Figure 3. The c-indexes of nomograms with TG/HDL-C were 0.732 and 0.725 for 5-year OS in the two cohorts, respectively. On the contrary, c-indexes of nomograms without TG/HDL-C predicting 5-year OS were 0.621 and 0.594, respectively. Additionally, the predictive accuracy of the nomogram with TG/HDL-C was significantly higher than that without TG/HDL-C in predicting 5-year OS for both two cohorts (all *P*<0.05, Figure 3).

## Discussion

In the present study, we observed that the predictive power of TG/HDL-C was markedly superior to other serum lipids through the ROC analysis, and high TG/HDL-C was significantly independent prognostic factor for worse OS by using the X-tile to determine the optimal threshold for the TG/HDL-C in a prospective cohort. In addition, TG/HDL-C could improve the prognostic nomogram to predict the 5-year OS of GC patients, which was further confirmed in the test cohort.

As we all know, it is important to distinguish risk factors for tumor progression and strengthen the surveillance to lower the rate of recurrence and improve the quality of life for tumor patients. Up to now, the diagnosis of GC relies on clinical and physical examination, such as gastroscope and histopathology. However, the application of tumor biomarkers has not played an important role in the screening, treatment, surveillance and prevention of GC 12. Plasma or serum reflects the biological object for tumor biomarkers as it represents physiological and pathological statuses 13. TG and HDL-C are usually considered as the important serum lipids, which are involved in energy storage, signal transduction, and structural composition. Furthermore, several studies have reported that serum lipids were associated with clinical outcomes in many types of malignancies including GC 4, 14. The present study enrolled the training and test cohorts of GC patients to explore the correlation between serum lipids and GC.

Hypertriglyceridemia is a common status of dyslipidemia, which is associated with cardiovascular and cerebrovascular diseases 15, 16. Furthermore, several epidemiological studies had addressed the correlation between serum lipids and cancer risk. A large-scale cohort study suggested that high TG increased risk of rectal cancer and breast cancer 17, 18. However, the inconsistent results of serum TG levels and GC risk have been reported from a FIESTA study 14 and a cohort study 19. The correlation between TG and GC remains unclear. In our study, we enroll two independent studies to confirm the correlation between serum lipids and GC. We observed that patients with TG≥1.2 had a worse OS than those with TG<1.2 in both two cohorts. Furthermore, it could improve the prognostic nomogram in the prediction of the survival of GC patients. However, the detailed biological mechanisms of them require further investigation.

With regard to HDL-C, mounting evidence showed that it presents anti-oxidative and anti-inflammatory properties. A precious study showed that HDL-C inhibited LDL-C oxidative damage to prevent lipid peroxidation 20. However, the association of HDL-C and cancer remained contradictory 4, 21. In our study, although we observed no significant difference between HDL-C and GC, it could improve predictive significance of nomogram in the prediction of the survival of GC patients.

The present study of two cohorts aimed to further confirm the prognostic significance of TG/HDL-C in GC patients. Meanwhile, prognostic nomogram including TG/HDL-C can efficiently predict the 5-year OS of GC patients. However, the individuals in our study are from the single center, and the sample size is small. Therefore, our findings should further be verified by other large sample and prospective studies from multi-center organizations.

In conclusion, our findings have demonstrated that preoperative TG/HDL-C was an efficient and independent prognostic factor to predict 5-year case fatality of GC patients and to improve the efficacy of its prognostic nomogram. It may be considered as a readily indicator to monitor GC progression and to optimize therapeutic strategies for the GC.

## Figures and Tables

**Table 1:** Baseline characteristics for training and test cohort patients.

Clinical characteristics	Training cohort 572 (%)	Test cohort 385	*P*
Age	65 (56-73)	64 (55-74)	0.452
Gender (male)	391 (68.4)	240 (62.3)	0.054
Smoking	125 (21.9)	78 (20.3)	0.554
Drinking	71 (12.4)	54 (14.0)	0.468
*Helicobator pylori*	302 (52.8)	195 (50.6)	0.514
Tumor differentiation (well/moderate/poor)	99/237/236	56/178/151	0.280
pT stage (1/2/3/4)	94/60/227/191	48/42/160/135	0.413
pN stage (0/1/2/3)	149/168/110/145	92/140/71/82	0.133
Metastasis	202 (35.3)	129 (33.5)	0.564
Chemotherapy	280 (49.0)	184 (47.8)	0.725
Curative/palliative	325/247	201/184	0.160
TG (mmol/L)	1.54±1.09	1.50±1.14	0.251
TC (mmol/L)	4.43±1.04	4.44±1.21	0.202
HDL-C (mmol/L)	1.06±0.38	1.09±0.44	0.064
LDL-C (mmol/L)	2.68±0.92	2.62±0.82	0.512
TG/HDL-C (mmol/L)	1.60±1.41	1.62±1.38	0.231

TG, triglyceride; TC, total cholesterol; HDL-C, high-density lipoprotein cholesterol; LDL-C, low-density lipoprotein cholesterol; TG/HDL-C, TG to HDL-C ratio. Values are medians (interquartile range) or frequencies and percentages. Patients received treatment for either curative or palliative purposes according to the Japanese Classification of Gastric Cancer Guidelines. Data were analyzed using χ^2^ test or Mann-Whitney *U* test.

**Table 2:** Associations between TG/HDL-C level and clinical characteristics in training and test cohort patients.

Variable	TG/HDL-C group					
	Training cohort			Test cohort		
	Low group 144	High group 428	*P*	Low group 71	High group 314	*P*
**Age**	63 (54-73)	66 (55-74)	0.028	62.5 (57-78)	65 (57-74)	0.013
**Gender**						
Male	92 (63.8)	299 (69.9)	0.183	51 (71.8)	189 (60.2)	0.068
Female	52 (36.1)	129 (30.1)		20 (28.2)	125 (39.8)	
**Smoking**						
Never	112 (77.8)	335 (78.3)	0.901	54 (76.1)	253 (74.8)	0.392
Yes	32 (22.2)	93 (21.7)		17 (23.9)	61 (25.2)	
**Drinking**						
Never	129 (89.6)	372 (86.9)	0.401	60 (84.5)	271 (86.3)	0.693
Yes	15 (10.4)	56 (13.1)		11 (15.5)	43 (13.7)	
***Helicobator pylori***						
Negative	59 (41.0)	211 (49.3)	0.083	32 (45.1)	158 (50.3)	0.424
Positive	85 (59.0)	217 (50.7)		39 (54.9)	156 (49.7)	
**Differentiation**						
Well	23 (16.0)	76 (17.8)	0.464	7 (9.9)	49 (15.6)	0.368
Moderate	66 (45.8)	171 (40.0)		37 (52.1)	141 (44.9)	
Poor	55 (38.2)	181 (42.3)		27 (38.0)	124 (39.5)	
**pT stage**						
T1-T2	74 (51.4)	80 (18.7)	<0.001	32 (45.1)	58 (18.5)	<0.001
T3-T4	70 (48.6)	348 (81.3)		39 (54.9)	256 (81.5)	
**pN stage**						
N0	28 (19.4)	116 (27.1)	0.067	18 (25.4)	74 (23.6)	0.750
N1-N3	116 (80.6)	312 (72.9)		53 (74.6)	240 (76.4)	
**Metastasis**						
Absent	84 (58.3)	286 (66.8)	0.065	44 (62.0)	212 (67.5)	0.371
Present	60 (41.7)	142 (33.2)		27 (38.0)	102 (32.5)	
**Chemotherapy**						
No	79 (54.9)	213 (49.8)	0.290	37 (52.1)	164 (52.2)	0.986
Yes	65 (45.1)	215 (50.2)		34 (47.9)	150 (47.8)	
**Treatments**						
Curative	77 (53.5)	248 (57.9)	0.349	31 (43.7)	170 (54.1)	0.110
Palliative	67 (46.5)	180 (42.1)		40 (56.3)	144 (45.9)	

TG, triglyceride; TC, total cholesterol; HDL-C, high-density lipoprotein cholesterol; LDL-C, low-density lipoprotein cholesterol; TG/HDL-C, TG to HDL-C ratio. Values are medians (interquartile range) or frequencies and percentages. Data were analyzed using χ^2^ test or Mann-Whitney *U* test.

**Table 3:** Univariate and multivariate analyses of prognostic significance of TG/HDL-C levels.

	Training cohort			Test cohort		
Variable	Univariate analysis	Multivariate analysis		Univariate analysis	Multivariate analysis	
	*P*	HR (95%CI)	*P*	*P*	HR (95%CI)	*P*
Age	0.015	1.13 (1.06-1.57)	0.001	0.021	1.21 (1.02-1.82)	0.045
Gender (Male)	0.301			0.358		
Smoking	0.540			0.165		
Drinking	0.308			0.607		
*Helicobator pylori*	0.814			0.274		
Differentiation	<0.001			0.001		
Well		Reference			Reference	
Moderate/Poor		1.24 (0.65-1.62)	0.268		1.54 (0.86-2.65)	0.259
pT stage	<0.001			<0.001		
T1-T2		Reference			Reference	
T3-T4		1.85 (1.12-3.88)	0.011		1.94 (1.10-3.01)	<0.001
pN stage	<0.001			<0.001		
N0		Reference			Reference	
N1-N3		1.92 (1.14-3.18)	<0.001		1.63 (1.10-3.05)	0.002
Metastasis	<0.001			<0.001		
Absent		Reference			Reference	
Present		1.94 (1.26-2.78)	<0.001		2.11 (1.41-3.05)	<0.001
Chemotherapy	0.415			0.208		
Treatments	<0.001			<0.001		
Curative		Reference			Reference	
Palliative		1.46 (0.94-1.89)	0.127		1.49 (0.92-2.59)	0.098
TG (mmol/L)	0.006	0.85 (0.51-0.94)	0.033	<0.001	0.78 (0.52-0.90)	0.040
TC (mmol/L)	0.138			0.756		
HDL-C (mmol/L)	0.002	0.92 (0.84-1.51)	0.614	0.054		
TG/HDL-C	<0.001	0.65 (0.32-0.92)	0.001	<0.001	0.64 (0.45-0.91)	0.004

TG, triglyceride; TC, total cholesterol; HDL-C, high-density lipoprotein cholesterol; LDL-C, low-density lipoprotein cholesterol; TG/HDL-C, TG to HDL-C ratio; HR, hazard ratio; CI, confidence interval.

## Abbreviations

TG: triglyceride
HDL-C: high density lipoprotein cholesterol
triglyceride-to-high density lipoprotein cholesterol ratio: TG/HDL-C
GC: gastric cancer
TNM: tumor-node-metastasis
TC: total cholesterol
LDL-C: low-density lipoprotein cholesterol
IQR: inter-quartile ranger
OS: overall survival
ROC: receiver operating characteristic
AUC: area under ROC curve
c-index: concordance index
